# Rebuilding the microenvironment of primary tumors in humans: a focus on stroma

**DOI:** 10.1038/s12276-024-01191-5

**Published:** 2024-03-05

**Authors:** Siwon Mun, Hyun Jin Lee, Pilnam Kim

**Affiliations:** 1 Department of Bio and Brain Engineering, KAIST, Daejeon, 34141 South Korea; 2 Institute for Health Science and Technology, KAIST, Daejeon, 34141 South Korea

**Keywords:** Cancer microenvironment, Cancer models

## Abstract

Conventional tumor models have critical shortcomings in that they lack the complexity of the human stroma. The heterogeneous stroma is a central compartment of the tumor microenvironment (TME) that must be addressed in cancer research and precision medicine. To fully model the human tumor stroma, the deconstruction and reconstruction of tumor tissues have been suggested as new approaches for in vitro tumor modeling. In this review, we summarize the heterogeneity of tumor-associated stromal cells and general deconstruction approaches used to isolate patient-specific stromal cells from tumor tissue; we also address the effect of the deconstruction procedure on the characteristics of primary cells. Finally, perspectives on the future of reconstructed tumor models are discussed, with an emphasis on the essential prerequisites for developing authentic humanized tumor models.

## Revolutionizing cancer research: the power of reconstructed tumor models

Cancer research usually focuses on cancer cells, but the importance of the tumor microenvironment (TME-the environment around a tumor) is becoming more recognized. The TME, especially the tumor stroma, significantly influences cancer cell behavior. The tumor stroma consists of different cell types like fibroblasts, macrophages, and endothelial cells. The interactions between these cells aid tumor growth and development. However, current research models often don’t accurately represent the TME’s complexity and diversity. This study suggests a new method to recreate the TME in a lab, by breaking down patient tumors and rebuilding them using components from those tumors. This could offer a more precise model for cancer research and personalized treatment development. The study also emphasizes the need for standard methods to extract primary cells from tumor tissues, to maintain their original features.

This summary was initially drafted using artificial intelligence, then revised and fact-checked by the author.

## Introduction

In cancer research, the primary focus has traditionally centered on neoplastic cells. However, the importance of the tumor microenvironment (TME) is receiving increasing amounts of attention. The concept of the TME is far from new; Stephen Paget’s seminal “seed and soil” hypothesis in the 1880s first posited that specific organs or ‘soils’ offer a more conducive environment for certain cancer cells or ‘seeds’ to flourish^1^. Cancer research has since validated and expanded upon this theory, illuminating the multifaceted role of the TME—especially the tumor stroma—in modulating the behavior of cancer cells.

The tumor stroma is composed of noncellular compartments, such as the extracellular matrix (ECM), and cellular compartments, including a wide range of invading and resident cells, such as fibroblasts, macrophages, endothelial cells, pericytes, adipocytes, and immune cells (T cells, B cells, natural killer cells (NK cells), and dendritic cells (DCs))^2^. The dynamic interactions between tumor cells and stromal cells contribute to tumor hallmarks^3^, for instance, sustained proliferative signaling^4–6^, evasion of growth suppressors^4,7,8^, immune evasion^4^, replicative immortality^9^, tumor-promoting inflammation^10–13^, activating invasion and metastasis^14,15^, enhanced vasculature^16,17^, genome instability^18,19^, resistance to cell death^20,21^, and deregulated cellular metabolism^22,23^. Given the importance of such tumor-stromal interactions, cancer research has shifted its emphasis to the TME, leading to the exploration of TME-targeted treatment approaches.

**Fig. 1:**
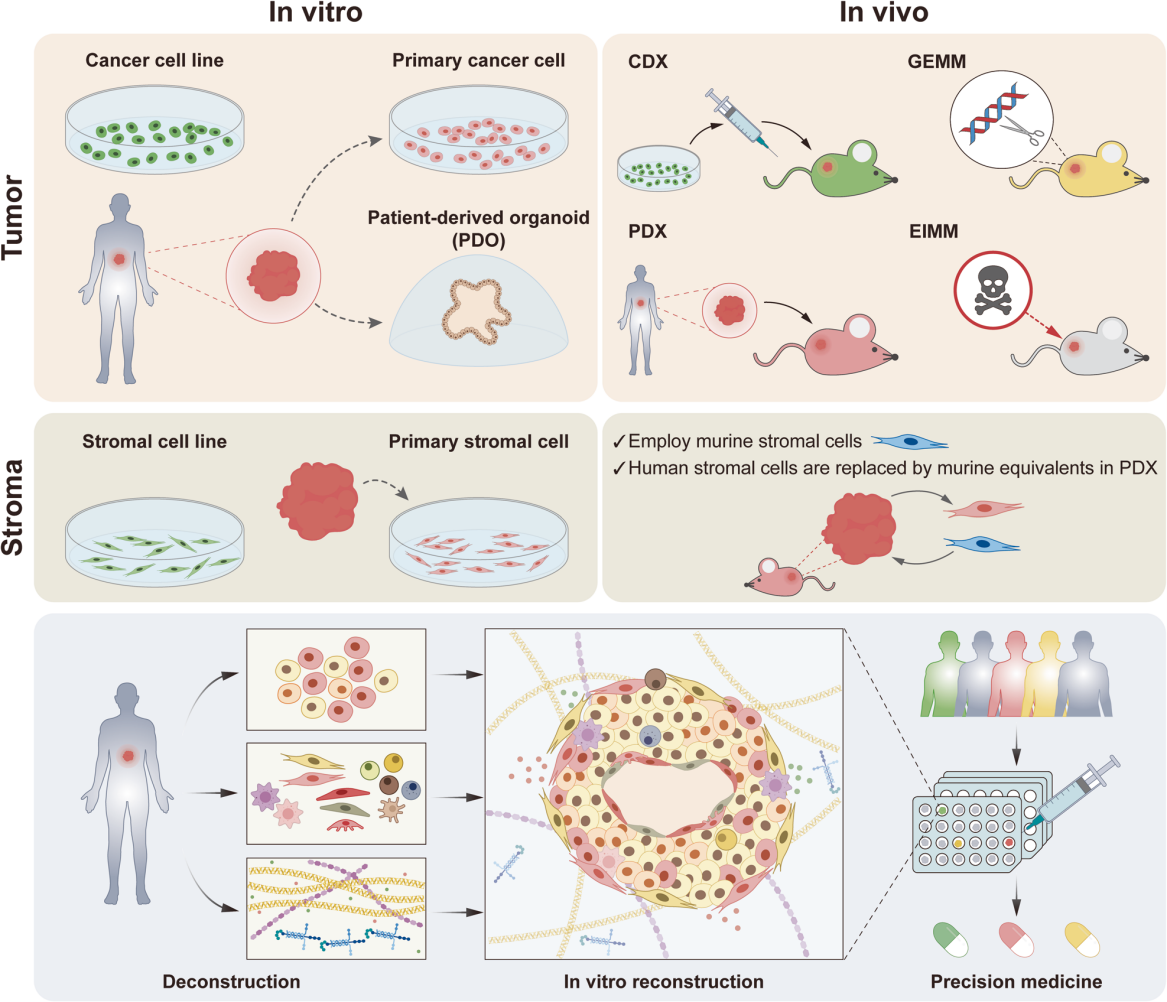
Sources employed for in vitro and in vivo tumor modeling. This figure outlines the major sources employed in tumor modeling. For in vitro representations of tumor cells, both established cancer cell lines and patient-derived tumor cells were used. Emerging models, such as patient-derived tumor organoids (PDOs), which are developed from patient-derived stem cells, serve to recapitulate the heterogeneity intrinsic to tumor cells more faithfully. In the context of in vitro stroma reconstruction, only stromal cell lines and primary stromal cells derived from tumor tissue can accurately represent the tumor stroma. For in vivo studies, mouse models are the most commonly utilized systems. These include cell line-derived xenograft (CDX) models that are generated by inoculating tumor cell lines into immunocompromised mice; patient-derived xenograft (PDX) models that are established through the implantation of patient tumor cells or tissue into immunocompromised mice; genetically engineered mouse models (GEMMs) that feature genomes altered to mimic in vivo genetic events; and environmentally induced mouse models (EIMMs) that are developed through the administration of carcinogens to immunocompetent mice. Notably, these models have limitations in their ability to incorporate a human stromal cell compartment, often relying on mouse stromal cells or substituting human stromal cells. Strategies focused on the deconstruction and subsequent reconstruction of the tumor microenvironment may offer more accurate representations of the heterogeneous tumor stroma.

With the emergence of TME-focused cancer research and the development of TME-targeting therapies, numerous attempts have been made to develop tumor models that incorporate the stroma (Fig. 1)^24–26^. Cancer cell lines and patient-derived tumor organoids (PDOs) are widely used in vitro tumor models to understand cancer biology and develop therapeutic targets. Although they are easy to maintain and amenable to high-throughput assays, they are limited by the exclusive cultivation of cancer cells^27^. To overcome this limitation, the incorporation of stromal cells is being pursued to recapitulate cancer cell-stromal cell interactions in vitro^28–30^. Although partial recapitulations of the tumor stroma affect the tumor cell phenotype and behavior, these processes involve only a few stromal cell types, and the translation of observations from in vitro to in vivo tumors requires further validation.

Animal models are alternatives for understanding human tumor biology and evaluating therapeutic responses as they provide an in vivo microenvironment. Mouse models, including genetically engineered mouse models (GEMMs), cell line-derived xenograft (CDX) models, environmentally induced mouse models (EIMMS), and patient-derived xenograft (PDX) models, are commonly used^31,32^. Among them, the PDX model, which is established by the implantation of patient-derived tumors, is preferred for studying human tumors because it represents the heterogeneity of original patient tumors and allows for the capture of complex tumor-stoma interactions^33^. However, the major concern with the PDX model is that the model adopts stromal components of mice. Even though human stroma is present after engraftment, it is rapidly lost and eventually replaced by mouse stroma, which alters tumor-stroma interactions^34,35^. The stroma replacement limits the applicability of mouse models in identifying human-specific mechanisms underlying stoma-driven tumor malignancy and assessing human-specific TME-targeting therapies. For these reasons, there is an urgent need to develop humanized in vitro models or in vivo model systems for studying human cancer.

One possible breakthrough toward developing a tumor model resembling the patient-specific TME involves deconstructing patient tumors and reconstructing them in vitro using components isolated from those tumors^36^. The stromal cell subpopulations and their proportions are unique to each patient, even within patients with the same type of cancer^37,38^. Therefore, dissecting patient tumors to identify the characteristics of stromal cell subpopulations and then utilizing them to construct tumor models could be beneficial for understanding intricate mutual interactions and advancing precision medicine.

Primary tumor cells and patient-derived organoids (PDOs) are predominantly used as in vitro models. Patient-derived primary tumor cells (PTCs) are preferred model systems for acquiring more reliable information for translating basic research to clinical application. The general primary cell isolation process can easily be utilized to obtain primary tumor epithelial cells^39^. In contrast, PDOs require specific cell sources, such as primary adult stem cells (ASCs) or pluripotent stem cells (PSCs), and a culture matrix, especially basement membrane extract^40^.

In addition to PTCs, stromal cells are isolated from tumor tissues to reconstruct the tumor niche. Although patient-derived primary stromal cells are already being extensively utilized in cancer research, most previous studies neglected to evaluate the subtypes of isolated stromal cells. For example, myofibroblastic cancer-associated fibroblasts (myCAFs), inflammatory CAFs (iCAFs), and antigen-presenting CAFs (apCAFs) are well-recognized CAF subtypes in pancreatic ductal cancer^41^. Each subtype of cancer has distinct effects^42^; however, many studies have used primary fibroblasts without considering CAF subtypes^43,44^. This subtype-neglecting approach could limit the understanding and recapitulation of stromal heterogeneity.

In this review, we provide an overview of the different types of stromal cells that have been identified and the current methods for isolating primary stromal cells from tumor tissues. We also summarize how the current isolation methods affect the primary stromal cell phenotype and key features. Finally, we discuss potential breakthroughs and considerations for overcoming the obstacles of conventional tumor models.

## Stromal cell heterogeneity in the tumor microenvironment

**Fig. 2:**
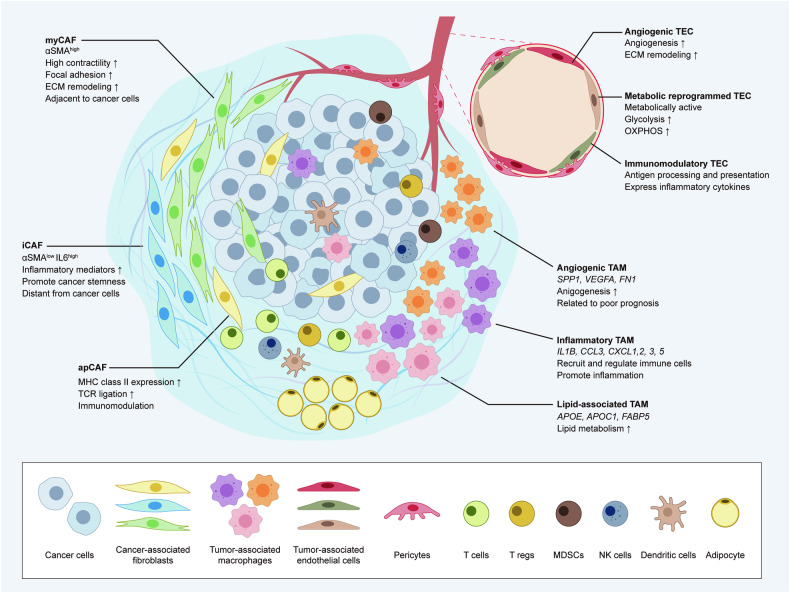
Heterogenous cell subpopulations in the tumor microenvironment. Schematic representation of TME heterogeneity. Several subpopulations of CAFs, TAMs, and TECs have been described, and the distinct markers and features of each cell type have been highlighted. The TME comprises various stromal cells in addition to tumor cells. Stromal cell heterogeneity is identified using cutting-edge technologies for single-cell analysis.

Many stromal cells make up the TME and actively engage with tumor cells, thus reshaping the tumor niche into a favorable niche for tumor progression. The stromal cells present in the TME include CAFs, tumor-associated macrophages (TAMs), tumor-associated endothelial cells (TECs), pericytes, adipocytes, and immune cells such as T cells, B cells, NK cells, DCs, and myeloid-derived suppressor cells (MDSCs) (Fig. 2)^2^. In Table 1, we summarize the three major types of stromal cells and outline the currently recognized subpopulations within each type.

**Table 1:** Previously identified stromal cell subpopulations in individual studies.

Stromal cell	Cancer type	Subpopulation	Markers	Feature	Ref
CAF	Breast cancer	CAF-S1	CD29^Med^ FAP^Hi^ FSP1^Low-Hi^ αSMA^Hi^ PDGFRb^Med-Hi^ CAV1^Low^	- High expression of CCL11, CXCL12, 13, 14 - Myofibroblastic subset - Enriched in TNBC - Immunosuppressive - Increase T lymphocyte survival and differentiation - Associated with accumulation of FOXP3 + T lymphocytes - Correlated with CD45+ hematopoietic cells and macrophages - Anti-correlated with CD8 + T lymphocytes - Enhance T_reg_ capacity to inhibit effector T cells	^136^
		CAF-S2	CD29^Low^ FAP^Neg^ FSP1^Neg-Low^ αSMA^Neg^ PDGFRb^Neg^ CAV1^Neg^	- Enriched in LumA BC	
		CAF-S3	CD29^Med^ FAP^Neg^ FSP1^Med-Hi^ αSMA^Neg-Low^ PDGFRb^Med^ CAV1^Neg-Low^	- Associated with Juxta-tumor	
		CAF-S4	CD29^Hi^ FAP^Neg^ FSP1^Low-Med^ αSMA^Hi^ PDGFRb^Low-Med^ CAV1^Neg-Low^	- Enriched in TNBC and HER2 BC - Myofibroblastic subset - Associated with CD8 + T lymphocytes - Anti-correlated with FOXP3 + T lymphocyte	
	Breast cancer	CAF-S1 ECM-myCAF	*GJB2, LRRC15*	- ECM-myofibroblastic CAF - Enriched in LumA BC - Involved in collagen synthesis and ECM organization	^38^
		CAF-S1 Detox-iCAF	*ADH1B, GPX3*	- Detoxification-inflammatory CAF - Enriched in TNBC - Involved in detoxification and inflammatory response	
		CAF-S1 IL-iCAF	*RGMA, SCARA5*	- Response to stimuli - Enriched in TNBC - Involved in the response to growth factor, TNF signaling, and IL pathway	
		CAF-S1 TGFβ-myCAF	*CST1, TGFB1*	- TGFβ-myofibroblastic CAF - Enriched in LumA BC - Involved in response to TGFβ stimulus and matrisome	
		CAF-S1 Wound-myCAF	*SEMA3C, SFRP4*	- Wound healing-myofibroblastic CAF - Enriched in LumA BC - Involved in the assembly of collagen fibrils and wound healing - Correspond to apCAF	
		CAF-S1 IFNγ-iCAF	*CCL19, CCL5*	- IFNγ and cytokines	
		CAF-S1 IFNαβ-iCAF	*IFIT3, IRF7*	- IFNαβ-inflammatory CAF	
		CAF-S1 Acto-myCAF	*GGH, PLP2*	- Actomyosin-myofibroblastic CAF	
		CAF-S2	FAP^Neg^ CD29^Low^ SMA^Neg^	- Abundant in healthy tissue	
		CAF-S3	FAP^Neg^ CD29^Med^ SMA^Neg^	- Abundant in healthy tissue	
		CAF-S4	FAP^Neg^ SMA^Hi^ CD29^Hi^ MCAM^Hi^	- Restricted to cancer and metastatic lymph nodes - Characterized by a perivascular signature - Pro-metastatic function - Contractile	
	Breast cancer	vCAF	*Rgs5*	- Vascular CAF - Upregulated vascular development and angiogenesis genes - Enriched in tumor core - Localized in proximity to vasculature	^51^
		mCAF	*Pdgfra, Mfap5, Dcn*	- Matrix CAF - Strong ECM signature - Upregulated ECM, matrisome, and EMT associated genes - Low abundance in the tumor core	
		cCAF	*Nuf2, Mki67*	- Cycling CAF - Upregulated cell-cycle-related genes - Proliferative segments of vCAF	
		dCAF	*Scrg1, Sox9, Sox10*	- Developmental CAF - Upregulated development and morphogenesis of tissue-associated genes - Originate from tumor cells that have undergone EMT	
	PDAC	myCAF	*ACTA2, TAGLN, MMP11, MYL9, HOPX, POSTN, TPM1, TPM2*, ACTA2^high^	- Myofibroblastic CAF - Adjacent to cancer cells - Associated with smooth muscle contraction, focal adhesion, ECM organization, and collagen formation	^137^
		iCAF	*IL6, PDGFRA, CXCL12, CFD, DPT, LMNA, AGTR1, HAS1, CXCL1, CXCL2, CCL2, IL8*, ACTA2^low^ Ly6C^high^	- Inflammatory CAF - High inflammatory mediators: *IL6, IL11, LIF* - Located in the desmoplastic areas of the tumor - Distant from cancer cells - Associated with the synthesis of hyaluronan and the complement pathway	
		apCAF	*H2-Ab1, Cd74, Saa3, Slpi*	- Antigen-presenting CAF - Express MHC class II-related genes - Induce TCR ligation in CD4 + T cells in an antigen-dependent manner	
	Lung cancer	Cluster 1	*COL10A1*	- Highly enriched in tumor - EMT-related signal - ECM phenotype - *HOXB2* and *FOXO1* are highly upregulated	^53^
		Cluster 2	*COL4A1*	- The highest expression of *ACTA2*, a myofibroblast marker - Involved in myogenesis, NOTCH pathway, and angiogenesis - Myogenesis phenotype	
		Cluster 4	*PLA2G2A*	- Similar to Cluster 1 - COL14A1^high^	
		Cluster 5	*MMP3*	- Low expression of myogenesis signature - High expression of mTOR signature and glycolysis genes	
		Cluster 6	*FIGF*	- Nonmalignant fibroblast - High expression of elastin - Low expression of some collagens: collagen type I, III, V, and VIII	
		Cluster 7	*CCL2*	- Present in NSCLC patients - Similar to Cluster 5 but with low expression of glycolysis genes	
	Gastric cancer	myCAF	*TPM1, TPM2, MYL9, TAGLN, POSTN*	- Myofibroblastic CAF - Prevalent in intestinal-type GC - Negatively correlated with tumor stemness	^138^
		iCAF	*IL6, IL11, IL24, CXCL1, CXCL2, CXCL5, CXCL6, MMP1, MMP3, MMP10*	- Inflammatory CAF - Prevalent in diffuse-type GC - Associated with GC invasion - Promote stemness of tumor cells, high stemness score	
		inCAF	*PDGFRA, POSTN, ID1, ID3*	- Intermediate CAF - Negatively correlated with tumor stemness - inCAF signal is increased with tumor progression from the premalignant state	
	Colorectal cancer	CAF-A	*FAP, MMP2, LUM, COL1A2*	- Involved in ECM remodeling - Intermediate state between NMFs and CAF-B	^139^
		CAF-B	*ACTA2, TAGLN, PDGFA*	- Express cytoskeletal genes known for activated myofibroblast markers	
		NMFs	*SFRP1/2, MFAP5, DPT, S100A4*	- Normal mucosa fibroblasts	
	Pancancer	myoCAF	*ACTA2, MYH11*	- Myofibroblastic CAF - Enriched tumorigenesis and myogenic regulons (TBX2, MEF2C each)	^48^
		inflaCAF	*MMP11, CTHRC1, FAP, TGFB1*	- Inflammatory CAF - Associated with dedifferentiation regulon	
		adiCAF	*CFD*	- Adipogenic CAF - Associated with EMT regulon	
		EndMT-CAF	*RGS5, ACTA2, PLVAP, VWF*	- Endothelial to Mesenchymal Transition CAF - Associated with angiogenesis and endothelial differentiation	
		PN-CAF	*MPZ, S100B, LGI4, PLP1*	- Peripheral nerve-like CAF	
		apCAF	*ACTA2, HLA-DRA, CD74, HLA-DRB1*	- Antigen-presenting CAF - Enriched in PDAC - Interaction with tumor-infiltrating T-cell clusters	
TAM	Breast cancer	Stromal Macrophage	CD11b + MRC1+	- Harbor potent T-cell activation capacity	^140^
		Hyperplastic Ductal Macrophage	CD11b-	- Associated with an advanced tumor stage - Potent phagocytes - Not efficient for activating CD8 T cells - Locally accumulated through the active proliferation	
		Malignant ductal TAM	CD11b + SPP1+	- Associated with poor prognosis - Regulate immunosuppressive functions of TAMs of monocytic origin	
	Breast cancer	LAM1:FABP5 TAM	*SPP1, FABP5*	- Similar to lipid-associated macrophages (LAM) - High expression of *TREM2* and lipid/fatty acid metabolic genes - Reduced proportion in HER2 + BC - Correlation with worse survival	^141^
		LAM2:APOE TAM	*APOE*	- Similar to lipid-associated macrophages - High expression of *TREM2* and lipid/fatty acid metabolic genes	
		CXCL10 TAM	*CXCL10, CXCL11*	- M1-like phenotype	
		EGR1, SIGLEC1 TAM	*IL2RA, CD209*	- M2-like phenotype	
	Gastric cancer	HSP + TAM	*HSPA6, HSPA1B, HSPB1*	- Increased HSP-associated genes	^142^
		THBS1 + TAM	*THBS1*	- N/A	
		Chemokine-TAM	*CCL3, CCL18, CCL20*	- Increased expression of chemokines	
		MMP-TAM	*MMP9, MMP12*	- MMP genes	
		Complement-TAM	*C1QA, C1QB, C1QC*	- Complement family	
		Cell cycle-TAM	*TOP2A, STMN1*	- Cell-cycle regulation genes	
	Colon cancer	C1QC + TAM	*C1Q genes, TREM2, MERTK, CD80*	- Derived from IL1B+ Tissue-resident macrophage (TRM) - Expression of *MAF/MAFB* and *JUN/FOS* - Increased inflammatory signatures, including complement activation, antigen processing, and presentation pathways	^59^
		SPP1 + TAM	*SPP1, MARCO, VEGFA*	- Derived from NLRP3 + TRM - Expression of the level of *HLA-DRs, CEBPB, and ZEB2* - Angiogenic signatures: enriched tumor angiogenesis, ECM receptor interaction, and tumor vasculature pathways	
	Colorectal cancer	C1QC + MRC1- TAM	*C1QC*	- Closely related to CD14/CD16 monocytes in blood	^60^
		SPP1 + TAM	*C1QC, MRC1, STAT1, PPARG*	- Tumor-specific filtration - Originated from THBS1 + TAM - Exhibit shorter progression-free survival	
		THBS1 + TAM	*THBS1*	- Promote malignant migration of cancer - Capable of performing antigen processing and presentation and regulating intestinal immune network for IgA production - Can differentiate into SPP1+ macrophages	
		VCAN + TAM	*VCAN*	- N/A	
	Colorectal cancer	Proinflammatory macrophage	*IL1B, IL6, S100A8, S100A9*	- Upregulation of genes associated with cytokines	^143^
		SPP1+ macrophage A	*SPP1, IL6*	- Enriched in tumor core and border - Proinflammatory phenotype - Association with CMS type 4	
		SPP1+ macrophage B	*SPP1, CD163, SEPP1, APOE, MAF*	- Enriched in tumor core and border - Anti-inflammatory phenotype	
		Proliferating macrophage	*MKI67, KIAA0101*	- Upregulation of genes associated with cell cycle	
	Hepatocellular carcinoma	TAM1	*FOLR2, CD163, C1QB, SEPP1*, CD163^high^ CD206^high^	- Fetal-like TAM - FOLR2 expressing TAM - Exhibit immunosuppressive interactions - Higher expression of immunomodulatory chemokines - Enrichment with TIGIT+ cells	^144^
		TAM2	*SPP1, TREM2, FABP5, NUPR1*, CD163^low^ CD206^low^	- SPP1 + TAM	
		TAM3	*MT1G, MT2A, MT1X*, CD163^low^ CD206^low^	- MT1G-enriched TAM	
	Pancancer	C1QC + TAM	*C1QC*	- Tumor enriched macrophage - Higher M2 signature and phagocytosis scores	^145^
		SPP1 + TAM	*SPP1*	- Tumor enriched macrophage - Higher M2 signature and angiogenesis signature	
		ISG15 + TAM	*ISG15*	- Tumor-enriched macrophage - Upregulated IFN inducible genes - Higher expression of canonical M1 signature	
		FN1 + TAM	*FN1*	- Tumor-enriched macrophage - Mainly present in kidney cancer - Proangiogenic TAM	
		INHBA + TAM	*INHBA*	- Compensate SPP1 + TAM in stomach cancer with a proangiogenic signature	
		VCAN + TAM	*VCAN*	- Compensate SPP1 + TAM in BC with a proangiogenic signature	
		LYVE1 Macrophage	*LYVE1*	- Identified within multiple cancer types - Enriched in noncancer tissue - Tissue-resident interstitial macrophage	
		NLRP3 Macrophage	*NLRP3*	- Enriched in noncancer tissue - Represent proinflammatory TRM (Tissue-resident macrophage)	
	Pancancer	HES1 + TAM	*C1QA, C1QB, C1QC, IGF1, CCL3, CCL4*	- Harbor an embryonic signature	^146^
		TREM2 + TAM	*APOC1, APOE, SPP1, FABP5*	- Accumulated only in tumor tissue - Involved in metabolic disorders - Potentially immunosuppressive role	
		IL4I1 + TAM	IL4I1 + PD-L1 + IDO1+ *CD38, IDO1, CXCL9, CXCL10, CXCL11*	- Antigen presentation, interaction with both Th2 and Th1 T cells, T-cell exhaustion, and tryptophan degradation - Suppress T cells and attract T_reg_s into the tumor by producing chemokine, expressing PD-L1 and PD-L2, and degrading IL4I1/AHR tryptophan - Exhibit immunosuppressive - Promote the entry of T_reg_ into the tumor	
		Proliferating TAM	*TOP2A, MKI67, IDO1*	- Accumulated in all cancer types	
TEC	PDAC	Endothelial 1	*IGFBP3, SPP1, CFH, IGLL5, TIMP1*	- Higher expression of *HIF1A* - Enriched for ECM organization, regulation of vasculature development, regulation of angiogenesis, cell junction assembly and epithelial cell migration	^147^
		Endothelial 2	*CLPS, PRSS1, CTRB1, CA4, CELA3A*	- Represent normal pancreatic tissue	
	Lung cancer	Cluster1	*MT2A*	- Normal EC	^53^
		Cluster 3	*IGFBP3*	- Tumor EC - Enrichment of Myc target, nucleotide metabolism, OXPHOS-associated genes - Immune activation-associated genes are downregulated	
		Cluster 4	*SPRY1*	- Tumor EC - Enrichment of Myc target, nucleotide metabolism, OXPHOS-associated genes - Immune activation-associated genes are downregulated	
		Cluster 5	*EDNRB*	- Normal EC	
		Cluster 6	*PDPN, PROX1*	- Lymphatic EC	
	Gastric cancer	E0	*IGFBP5, STC1, IGFBP3*	- Influence angiogenic sprouting - Upregulation of mTOR and IGF-1 signaling - Increase the invasion and migration of tumor cells	^148^
		E1	*FOXO1, FOXP1, JUN*	- Associated with the regulation of T-cell exhaustion signaling pathway - Suppress immune response	
		E2	N/A	- Low activity - Normal endothelial cells	
		E3	*NRP1, FGFR1*	- VEGF receptor encoding genes are significantly upregulated - White adipose tissue browning pathway and STAT3 pathways are activated - Promote cancer cell invasion and angiogenesis	
	Gastric cancer	Endo1	*COL4A1, COL4A2, PROS1*	- Predominantly enriched in tumor - Downregulated MHC class II genes - Limited antigen presentation function - Strong activation of TNF, VEGF, PDGF, PGF, and Notch signaling - Involved in angiogenesis	^149^
		Endo2, Endo3, Endo4	N/A	- N/A	
	Colorectal cancer	Tip-like EC	*RGCC, RAMP3*	- Overrepresentation of regulators of angiogenesis in tumor - Overrepresentation of antigen processing and presentation in normal	^143^
		Stalk-like EC	*ACKR1, SELP*	- Associated with apoptosis inhibition and proliferation	
		Proliferative EC	*BIRC5, CKS1B*	- Overexpression of *BIRC5* and *CKS1B*	
		Lymphatic EC	*LYVE, PROX1*	- Found both in normal and tumor	
	Hepatocellular carcinoma	PLPP3 + TEC	*PLPP3*	- Enriched in tumor tissue	^144^
		PLVAP + TEC	*PLVAP, HLA-DRA*,	- Enriched in tumor tissue - Facilitate the emergence of fetal-like macrophages - Mainly enriched in fetal and tumor tissues - Major subset expressing the receptor for VEGF	
		IGFBP3 + TEC	*IGFBP3*	- Enriched in tumor tissue	
	Clear cell renal cell carcinoma	AVR1 TEC	*PLVAP, FLT1, KDR, FLT4, EDNRB, VWF, HSPG2*	- Higher expression of VEGF receptor - Upregulation of genes involved in hemostasis, angiogenesis, and stimulation of endothelial growth and regeneration	^63^
		AVR2 TEC	*ACKR1, SELP*	- Evade angiogenesis inhibitors	
	Glioblastoma	Pe1 EC	*KLF2, TIMP3, SLC2A1, SLCO1A2, TSC22D1, DEGS2, CAVIN2*	- Quiescent endothelial cells derived from nonmalignant tissue - Associated with vascular integrity and BBB function	^150^
		Co1 EC	*COL4A1, COL4A2, HSPG2, INSR, KDR*	- Derived from tumor core - Angiogenic phenotype - Associated with developmental and tumor angiogenesis, vascular basement membrane remodeling, cytoskeletal rearrangements, angiogenic sprouting, and endothelial tip cell formation	
		Co2 EC	*TMSB4X, RPLP2, RPL39, GAPDH, VIM, ACTB*	- Derived from tumor core - Intermediate phenotype - Associated with cytoskeletal and ribosomal protein expression	
		Pe2 EC	*CCL3, CCL4, CCL4L2, HLA-DRB1, HLA-DRA, HLA-DPA1, HLA-DPB1, HLA-DQB1*	- Immune-activated phenotype derived from nonmalignant tissue - Expression of inflammatory cytokines and MHC II-mediated antigen presentation genes	
		Co3 EC	*NR4A3, IL1B, IL1R1, SELE, SELP, VACM1*	- Derived from tumor core - Upregulation of immune-activated genes - Associated with inflammation and immune cell recruitment	
	Pancancer	ESM1 tip EC	*ESM1, NID2*	- Only resided in malignant tissue - Upregulation of glycolysis and OXPHOS	^37^
		ACKR1^high^ HEV and venous EC	*ACKR1, SELP*	- ACKR1^high^ endothelial venules and venous EC - Enriched in tumor	
		CA4 capillary EC	*CA4, CD36*	- Characterized by PLVAP and IGFBP7	
		FBLN5 arterial EC	*FBLN5, GJA5*	- Upregulated fatty acid biosynthesis	
		PROX1 lymphatic EC	*PROX1, PDPN*	- Increased fatty acid oxidation	
		TECs	*PLVAP, IGFBP7*	- Activation of HOXB pathways - Reduced carbonic acid metabolism	

*AVR* aortic valve replacement, *BBB* brain‒blood barrier, *CMS* consensus molecular subtype, *EMT* epithelial–mesenchymal transition, *GC* gastric cancer, *HER2, BC* Her2-positive breast cancer, *LumA, BC* luminal A breast cancer, *NMF* normal mucosa fibroblasts, *NSCLC* non-small cell lung cancer, *OXPHOS* oxidative phosphorylation, *PDAC* pancreatic ductal adenocarcinoma, *TNBC* triple-negative breast cancer, *TRM* tissue-resident macrophage. *ACKR1* atypical chemokine receptor 1 (Duffy blood group), *ACTA2* actin alpha 2, smooth muscle, *ACTB* actin beta, *ADH1B* alcohol dehydrogenase 1B (class I), beta polypeptide, *AGTR1* angiotensin II receptor type 1, *AHR* aryl hydrocarbon receptor, *APOC1* apolipoprotein C1, *APOE* apolipoprotein E, *ASMA* actin alpha 1, skeletal muscle, *BIRC5* baculoviral IAP repeat containing 5, *C1QC* complement C1q C chain, *CA4* carbonic anhydrase 4, *CAV1* caveolin 1, *CAVIN2* caveolae associated protein 2, *CCL2* C-C motif chemokine ligand 2, *CCL4L2* C-C motif chemokine ligand 4 like 2, *CD4* CD4 molecule, *CEBPB* CCAAT enhancer binding protein beta, *CELA3A* chymotrypsin like elastase 3A, *CFD* complement Factor D, *CFH* complement Factor H, *CKS1B* CDC28 protein kinase regulatory subunit 1B, *CLPS* colipase, *COL10A1* collagen type X alpha 1 chain, *COL14A1* collagen type XIV alpha 1 chain, *COL4A1* collagen type IV alpha 1 chain, *COL4A2* collagen type IV alpha 2 chain, *CST1* cystatin SN, *CTHRC1* collagen triple helix repeat containing 1, *CTRB1* chymotrypsinogen B1, *CXCL1* C-X-C motif chemokine ligand 1, *Dcn* Decorin, *DEGS2* delta 4-desaturase, sphingolipid 2, *DPT* dermatopontin, *EDNRB* endothelin receptor type B, *EGR1* early growth response 1, *ESM1* endothelial cell specific molecule 1, *FABP5* fatty acid binding protein 5, *FAP* fibroblast activation protein alpha, *FBLN5* fibulin 5, *FGFR1* fibroblast growth factor receptor 1, *FIGF* vascular endothelial growth factor D, *FLT1* Fms related receptor tyrosine kinase 1, *FN1* fibronectin 1, *FOLR2* folate receptor beta, *FOS* Fos proto-oncogene, AP-1 transcription factor subunit, *FOXO1* forkhead Box O1, *FOXP1* Forkhead Box the P1, *FSP1* S100 calcium binding protein A4, *GAPDH* glyceraldehyde-3-phosphate dehydrogenase, *GGH* gamma-glutamyl hydrolase, *GJA5* gap junction protein alpha 5, *GJB2* gap junction protein beta 2, *GPX3* glutathione peroxidase 3, *H2AB1* H2A. B variant histone 1, *HAS1* hyaluronan synthase 1, *HER2* Erb-b2 receptor tyrosine kinase 2, *HES1* Hes family bHLH transcription Factor 1, *HIF1A* hypoxia inducible factor 1 subunit alpha, *HLA-DPB1* major histocompatibility complex, class II, DP beta 1, *HLA-DQB1* major histocompatibility complex, class II, DQ beta 1, *HLA-DR* human leukocyte antigen - DR isotype, *HLA-DRA* major histocompatibility complex, class II, DR alpha, *HLA-DRB1* major histocompatibility complex, class II, DR beta 1, *HOPX* HOP homeobox, *HOXB* homeobox B, *HOXB2* Homeobox B2, *HSPG2* Heparan sulfate proteoglycan 2, *ID1* Inhibitor of DNA binding 1, *IDO1* indoleamine 2,3-dioxygenase 1, *IFIT3* interferon induced protein with tetratricopeptide repeats 3, *IGF1* insulin like growth factor 1, *IGFBP3* insulin like growth factor binding protein 3, *IGLL5* immunoglobulin lambda like polypeptide 5, *IL1B* interleukin 1 beta, *IL1R1* interleukin 1 receptor type 1, *IL2RA* interleukin 2 receptor subunit alpha, *IL4I1* interleukin 4 induced 1, *INHBA* inhibin subunit beta A, *INSR* insulin receptor, *IRF7* interferon regulatory factor 7, *ISG15* ISG15 ubiquitin like modifier, *JUN* Jun proto-oncogene, AP-1 transcription factor subunit, *KDR* kinase insert domain receptor, *KIAA0101* PCNA clamp associated factor, *KLF2* KLF transcription factor 2, *LGI4* leucine rich repeat LGI family member 4, *LIF* LIF interleukin 6 family cytokine, *LMNA* lamin A/C, *LRRC15* leucine rich repeat containing 15, *Ly6C* lymphocyte antigen 6 family member C 1, *LYVE1* lymphatic vessel endothelial hyaluronan receptor 1, *MAF* MAF bZIP transcription factor, *MAFB* MAF bZIP transcription factor B, *MARCO* macrophage receptor with collagenous structure, *MEF2C* myocyte enhancer Factor 2C, *MERTK* MER proto-oncogene, tyrosine kinase, *Mfap5* microfibril associated protein 5, *Mki67* antigen identified by monoclonal antibody Ki 67, *MMP1* matrix metallopeptidase 1, *MPZ* myelin protein zero, *MRC1* mannose receptor C-type 1, *MT1B* metallothionein 1B, *MT1X* metallothionein 1X, *MT2A* metallothionein 2A, *MYH11* myosin heavy chain 11, *MYL9* myosin light chain 9, *NID2* nidogen 2, *NLRP3* NLR family pyrin domain containing 3, *NR4A3* nuclear receptor subfamily 4 group A member 3, *NRP1* neuropilin 1, *Nuf2* NUF2, NDC80 kinetochore complex component, *NUPR1* Nuclear protein 1, transcriptional regulator, *PDGF* platelet-derived growth factor, *Pdgfra* platelet derived growth factor receptor alpha, *PDGFRB* platelet derived growth factor receptor beta, *PD-L1* CD274 molecule, *PDPN* podoplanin, *PGF* placental growth factor, *PLA2G2A* phospholipase A2 group IIA, *PLP1* proteolipid protein 1, *PLPP3* phospholipid phosphatase 3, *PLVAP* plasmalemma vesicle-associated protein, *POSTN* periostin, *PPARG* peroxisome proliferator activated receptor gamma, *PROS1* protein S, *PROX1* prospero homeobox 1, *PRSS1* serine protease 1, *RAMP3* receptor activity modifying protein 3, *RGCC* regulator of cell cycle, *RGMA* repulsive guidance molecule BMP coreceptor a, *Rgs5* regulator of G-protein signaling 5, *RPLP2* Ribosomal protein lateral stalk subunit the P2, *S100A8* S100 calcium binding protein A8, *S100B* S100 calcium binding protein B, *Saa3* serum amyloid A 3, *SCARA5* scavenger receptor class A member 5, *Scrg1* scrapie responsive gene 1, *SELE* selectin E, *SELP* selectin P, *SEMA3C* semaphorin 3C, *SEPP1* selenoprotein P, *SFRP4* secreted frizzled related protein 4, *SIGLEC1* sialic acid binding Ig like lectin 1, *SLC2A1* solute carrier family 2 member 1, *SLCO1A2* solute carrier organic anion transporter family member 1A2, *Slpi* secretory leukocyte peptidase inhibitor, *Sox9* SRY (sex determining region Y)-Box 9, *SPP1* CXXC finger protein 1, *SPRY1* Sprouty RTK signaling antagonist 1, *STAT1* signal transducer and activator of transcription 1, *STC1* stanniocalcin 1, *TAGLN* transgelin, *TBX2* T-box transcription factor 2, *TFF3* Trefoil factor 3, *TGFB1* transforming growth factor beta 1, *THBS1* thrombospondin 1, *TIGIT* T-cell immunoreceptor with Ig and ITIM domains, *TIMP1* TIMP metallopeptidase inhibitor 1, *TMSB4X* thymosin beta 4 X-linked, *TNF* tumor necrosis factor, *TOP2A* DNA topoisomerase II alpha, *TPM1* tropomyosin 1, *TREM2* triggering receptor expressed on myeloid cells 2, *TSC22D1* TSC22 domain family member 1, *VACM1* Cullin 5, *VCAN* versican, *VEGFA* vascular endothelial growth Factor A, *VIM* Vimentin, *VWF* Von Willebrand factor, *ZEB2* Zinc finger E-box binding homeobox 2.

### Cancer-associated fibroblasts (CAFs)

CAFs are the most abundant stromal cell type within the TME. For decades, CAFs were considered as a single homogenous population. In addition, definitive markers of CAFs were absent^45^. This lack of awareness about CAFs led to conflicting outcomes in previous CAF research. For example, S100 calcium binding protein A4 (S100A4) is a known marker of CAFs. Vascular endothelial growth factor A (VEGF-A) and tenascin-C (TNC), which are produced by S100A4-positive CAFs (S100A4+ CAFs), promote tumor metastasis^46^. However, in another study, S100A4+ CAFs contributed to tumor suppression by depositing collagen around carcinogens and preventing DNA damage to epithelial cells^47^. These findings further obscure the comprehensive understanding of the distinctive attributes of CAFs. Recent studies have revealed that CAFs exist in different compositions depending on the tumor and patient, and these CAFs have different molecular characteristics^38,48^. At the pancancer level, various CAF subsets were observed. For instance, myoCAFs, inflaCAFs, and apCAFs were discovered via pancancer analyses, and each share similar transcriptomic patterns of myCAFs, iCAFs, and apCAFs identified in pancreatic ductal adenocarcinoma (PDAC)^48^. In PDAC, three major types of CAFs were identified. myCAFs constitute the majority of CAFs in tumor samples and express a high level of αSMA. This subtype is characterized by the significant expression of genes related to smooth muscle contraction, focal adhesion, and ECM remodeling, these genes are conventionally regarded as phenotypes of activated fibroblasts. In contrast, iCAFs are characterized by the increased expression of inflammatory factors such as IL6, IL8, and chemokines. apCAFs are a distinct CAF subtype from myCAFs and iCAFs, and they express high levels of MHC class II family-associated genes^49^.

Furthermore, the spatial distribution of CAF subsets within the TME adds to their heterogeneity^50^. For example, myCAFs and iCAFs are differentially distributed in the tumor niche of PDAC. myCAFs are located adjacent to tumors, while iCAFs are located at a much farther distance from tumors^25^. Bartoschek et al. also confirmed the distinct location of CAF subsets in breast cancer. Vascular CAFs (vCAFs) exhibit angiogenic signatures and are localized near the vasculature and tumor core. Unlike vCAFs, matrix CAFs (mCAFs) with strong ECM signatures are found at low levels in the tumor core^51^. Therefore, to develop in vitro tumor models with high-fidelity, the spatial distribution of stromal cells should be considered because spatial intercellular communication between tumor cells and stromal cells occurs and impacts the prognosis and response to antitumor therapies.

### Tumor-associated macrophages (TAMs)

TAMs are generally regarded as contributors to tumor progression due to their involvement in ECM remodeling, angiogenesis, and immunosuppression^52^. These functions foster the development of a microenvironment conducive to tumor growth. However, recent research highlights the fact that TAMs also have antitumor activities that restrain tumor progression and improve patient prognosis^53,54^. The inherent plasticity of macrophages allows them to sense and adapt to cues from the microenvironment, which leads to altered macrophage phenotypes related to tumor-promoting or tumor-restraining functions^55^.

Macrophages have been classified into two polarized states: M1 and M2. M1 macrophages are characterized by a classically activated phenotype triggered by interferon-gamma (IFN-γ) or lipopolysaccharide (LPS). M1 macrophages exhibit proinflammatory properties. In contrast, M2 macrophages are in an alternatively activated state. M2 macrophages are induced by certain cytokines, such as IL-4, IL-10, and IL-13, and exhibit anti-inflammatory phenotypes^56^.

However, this conventional classification system oversimplifies the diverse nature of macrophages^57,58^. The diverse states of TAMs are being continuously identified (Table 1). For example, Zhang et al. classified TAMs in colon cancer into two main subsets: complement C1q C chain-positive TAMs (*C1QC* + TAMs) and secreted phosphoprotein 1-positive TAMs (*SPP1* + TAMs). *SPP1* + TAMs were characterized by increased expression of *SPP1*, macrophage receptor with collagenous structure (*MARCO*), and vascular endothelial growth factor A (*VEGFA*) and were associated with tumor angiogenesis. Signatures associated with colorectal adenoma and metastatic liver cancer pathways were also increased in this subset, which implies that the *SPP1*+ subset may possess protumorigenic and prometastatic functions in colon cancer^59^. In a separate study on colorectal cancer (CRC), *SPP1* + TAMs were also recognized as a tumor-promoting subset. Among the four subpopulations of TAMs with unique markers, *SPP1* + TAMs are a specific subset that infiltrates tumors, and CRC patients with a greater infiltration of this subset have a poor prognosis^60^. Tu et al. discovered a unique macrophage subset in osteosarcoma. *C1Q* + TAMs were identified in patients with high levels of immune infiltration. Patients with high levels of immune infiltration had an improved prognosis compared to those with low levels of immune infiltration. Although *C1Q* + TAMs are a tumor-restraining subset, they do not specifically express M1 or M2 signature genes^54^. Likewise, recent research has highlighted the existence of protumor and antitumor TAM subsets beyond the conventional M1 and M2 classifications. The distribution and characteristics of TAM subsets vary according to their origins and specific tumor microenvironments^58^. For a more precise representation of heterogeneous TAMs in vitro, a comprehensive understanding of TAM subsets and their biology is crucial. Such insights enable the construction of a more authentic TME, thereby facilitating studies on the subset-specific polarization of TAMs.

### Tumor endothelial cells (TECs)

Multiple studies have recently revealed the presence of TEC heterogeneity at the single-cell level^61,62^. Heterogeneous subsets of TECs have been identified across various organs, and each subset may have a distinct molecular phenotype and function. For example, renal cell TECs are classified into two distinct subsets: the AVR-1 and AVR-2 groups^63^. The AVR-1 subset is positive for plasmalemma vesicle-associated protein (*PLVAP)*, von Willebrand factor (*VWF*), heparan sulfate proteoglycan 2 (*HSPG2*), and endothelin receptor type B (*EDNRB*), while the AVR-2 subset is characterized by atypical chemokine receptor 1 (*ACKR1*) and selectin P (*SELP*). The two subsets differ not only in molecular expression but also in clinical benefit. *PLVAP* + ACR-1 was predominantly abundant in tumor tissues and negatively correlated with patient survival. However, ACKR + ACR-2 cells exhibited decreased expression of VEGF receptors, such as kinase insert domain receptor (*KDR*) and fms-related receptor tyrosine kinase 1 (*FLT1*), indicating that this subset may contribute to antiangiogenic therapy (AAT) evasion^63^. As demonstrated in previous studies, TECs are key stromal cells in the TME, and they can predict therapeutic response. Although many researchers have struggled to develop vascularized tumor models and mimic the near-native features of solid tumors in vitro, most studies have only focused on structural reconstruction of the microvasculature^64,65^. By incorporating affiliated TEC subsets and reconstructing microvessels in tumor models, it would be possible to generate high-fidelity tumor models.

## General process of primary cell isolation and expansion

**Fig. 3:**
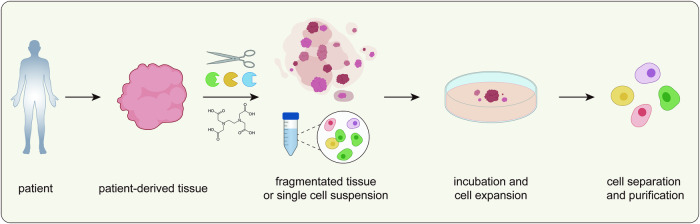
General isolation process for primary cells. A schematic illustration of the primary cell isolation process. Mechanical, enzymatic, and chemical methods can be utilized to dissociate tumor tissue acquired from patients. The tissue is divided into small pieces or single-cell suspensions, followed by incubation and expansion of the primary cells. Finally, the target cells are purified from the cell mixtures and utilized for downstream processes.

Unlike peripheral blood cells, which can be readily isolated by centrifugation or magnetic beads^66,67^, tissue-embedded cells require more complex procedures to isolate the desired cell types. The general procedure for isolating and cultivating primary cells from solid tissue involves five steps: tissue acquisition, dissection, tissue disaggregation, incubation/cell expansion, and cell separation or purification (Fig. 3). Here, we summarize the purpose of each step and the methods currently employed in the process (Table 2).

**Table 2:** Isolation methods for various stromal cells in individual studies.

Cell/ Cancer type	Dissociation		Incubation		Purification/Identification	Ref
	Mechanical	Chemical/Enzymatic	Media	Substrate		
Cancer-associated fibroblast (CAF)						
Head and neck cancer	*Not used*	10 mg/ml Collagenase P (2 hr, 37 °C)	Fibroblast growth medium, 20% FCS, 1 ng/ml BFGF, 5 µg/ml Insulin, 1% P/S, 1% Gentamycin	Tissue culture flask	- VIM + αSMA + CD45- CD68- MelanA- HMB45- KRT- CD34- - Identified by flow cytometry and immunocytochemistry	^93^
Melanoma	- Mincing - Scalpel - 1–4 mm^2^	0.25% Trypsin + 0.02% EDTA (20 min, 37 °C)	High glucose DMEM, 10% FBS, 100U/ml penicillin, 100 µg/ml streptomycin, 100 µg/ml gentamycin	6 well plate	- Fibroblast: VIM - Myofibroblast: αSMA - Immunofluorescence - Minimize keratinocyte contamination by earlier detachment of fibroblasts after trypsin treatment	^78^
Breast cancer	- Dissection	*Not used*	DMEM, 20% FBS, 1% Nonessential amino acid	Cell culture plate	- Spidle shaped morphology - Fibroblasts outnumbered tumor cells, and epithelial cells disappeared after passage 3	^151^
Gastric cancer	- Mincing - Scissors, scalpel - 2–4 mm	Enzyme H, R, A of tumor dissociation kit (from Miltenyi Biotec)	RPMI1640, 10% FBS	Cell culture dish	- CD90 - Morphology and FACS	^152^
Colorectal cancer	- Mincing - 1 mm	1 mg/ml Collagenase type I (1 h, 37 °C)	EGM2-MV	Tissue culture flask	- Proteomic and secretome analysis	^153^
Colorectal cancer	- Mincing - 1 mm^3^	1.5 mg/ml Collagenase IV + 20 µg/ml Hyaluronidase (1 h, 37 °C)	DMEM, 10% FBS	Cell culture dish	- 40 µm cell strainer - Red blood cell lysis buffer treatment - Wash away nonadherent cells after 3 h incubation - αSMA, VIM, and FAP - Immunofluorescence	^154^
Pancreatic cancer	- Mincing - Scissors, scalpel	*Not used*	DMEM, 10% FBS, 1X Glutamax, 1X P/S	12 well plate	- αSMA, VIM - myCAF: αSMA - iCAF: IL6 - apCAF: MHCII - Immunofluorescence	^155^
Liver cancer	- Cross sectioning	Enzyme H, R, A of tumor dissociation kit (from Miltenyi Biotec)	DMEM/F12 (1:1), 10% FBS	Cell culture dish	- FAP + αSMA + EpCAM- ECad- PECAM1- - Spindle-shaped morphology and FACS	^156^
Ovarian cancer	- Mincing - Scissors, scalpel - 3–5 mm^2^	- 0.25% Trypsin + 0.1% EDTA (30 min, 37 °C) - 0.5 mg/ml Hyaluronidase + 3 mg/ml Collagenase type 3 (6 h, 37 °C)	RPMI1640, 20% FBS, 100 U/ml penicillin, 100 µg/ml streptomycin	Cell culture dish	- αSMA, VIM, FAP, PDGFRα, Desmin, DDR2, S100A4 - Mitotic figure - Immunofluorescence	^157^
Tumor-associated macrophage (TAM)						
Breast cancer	- Chopping - razor blade	14 U/ml LiberaseTL + 28U/ml LiberaseDL + 15 mg/ml DNase I (1–18 h, 37 °C)	No cultivation	No cultivation	- 100 µm cell strainer - CD45 + CD11b + CD14 + CD163 + CD3- CD19- CD56- - FACS	^158^
Breast cancer	- Mincing - Scalpel - 2–4 mm	0.1% Collagenase type I + 0.2% Dispase type I + 1% DNase I (30 min, 37 °C)	DMEM, 10% FBS, 100 units/ml penicillin, 10 µg/ml streptomycin	96 well plate	- 70 µm cell strainer - Red blood cell lysis buffer treatment - CD11b + F4/80+	^159^
Breast cancer	- Mincing	LiberaseDH + DNase I (2 h, 37 °C)	DMEM, 5% FBS, 100 units/ml penicillin, 100 µg/ml streptomycin	24 well plate	- CD11b + F4/80+	^160^
Breast cancer	Not used	Collagenase IV + Hyaluronidase + Dispase II + DNase IV (15 min, 37 °C)	DMEM	Cell culture plate	- Isolate CD11b + Ly6G- cells	^161^
Breast cancer	- Mincing - razor blade	0.29 U/ml LiberaseDL + 0.56 U/ml LiberaseTL + 150 µg/ml DNase I (45 min, 37 °C)	*No cultivation*	*No cultivation*	- 100 µm cell strainer - Red blood cell lysis buffer treatment - DAPI- CD45 + CD11b + CD14 + CD163 + CX3CR1 + HLA-DR + CD3- CD19- CD56- - FACS	^79^
Mammary carcinoma	- Cutting, mincing - razor blade	1X Collagenase + 1X Hyaluronidase + 10U/ul DNase I (45 min, 37 °C)	RPMI, 20% FBS, 2 mM L-glutamine, 2 mM Sodium pyruvate, 1X Penicillin‒streptomycin, 55 µM 2-mercaptoethanol, 10 ng/ml mouse M-CSF	10 µg/ml Fibronectin-coated glass bottom plate	- Isolate CD11b+ cells by MACS - Determine purity of F4/80 + CD45+ cells by FACS	^91^
Colorectal cancer	- Slicing	Accumax (2 h, 37°C)	DMEM, 10% FBS	6 well plate	- CD68	^162^
Ovarian cancer	*Not mentioned*	*Not mentioned*	RPMI 1640, 10% FBS, 100 U/ml penicillin, 100 U/ml streptomycin	Cell culture plate	- Isolate CD14+ cells by MACS - Identified with CD206^high^ HLA-DR^low^ by FACS	^163^
Mouse solid tumor	- Mincing - Scissors, scalpel - 1–1.5 mm	10 U/ml Collagenase I + 400 U/ml Collagenase IV + 30 U/ml DNase I (25 min, 37 °C)	RPMI 1640, 10% FCS, 300 µg/ml L-glutamine, 100 U/ml penicillin, 100 µg/ml streptomycin, 0.02 mM beta-mercaptoethanol, 1 mM sodium pyruvate, 1 mM nonessential amino acids	*No cultivation*	- 70 µm filter - Erythrocyte lysis buffer treatment - CD11b + Ly6G- SiglecF- Ly6C^low^ - MACS, FACS	^95^
Tumor endothelial cell (TEC)						
Mammary cancer	- Mincing - Scissors - 5 mm	2 mg/ml Collagenase + 1 mg/ml Dispase + 1 mg/ml DNase (75 min, 37 °C)	Low glucose DMEM, 10% FBS, 10% Nu-Serum IV, 1% antibiotic-antimycotic, hFGF, VEGF, hEGF, R3-IGF-1, heparin	0.5% Gelatin-coated cell culture dish	- Scrape off nonspecific cells surrounding the identified EC colonies - Isolate CD31+ cells by MACS - Identified with CD31+ by immunofluorescence and FACS	^94^
Melanoma	- Cutting - Scalpel - 1 mm^3^	10 mg/ml Collagenase type II + 25 µg/ml DNase I (30 min, 37 °C)	Endothelial cell growth medium MV2	Collagenase type I- coated cell culture dish	- Density gradient centrifugation - Isolate CD31+ cells by MACS - Determine the purity and sort cells with CD31 + CD146+ by FACS	^164^
Melanoma	- Cutting - Scissors - 5 mm^3^	Collagenase type II + Collagenase type IV + DNase I (30 min, 37 °C)	*No cultivation*	*No cultivation*	- 70 µm cell strainer - Isolate CD45- EpCAM- CD31+ cells by MACS	^165^
Melanoma	- Mincing	Collagenase II	EGM-2MV, 15% FBS	1.5% gelatin-coated cell culture dish	- Remove blood cells by single sucrose step-gradient centrifugation - Isolate CD31+ cells by MACS - Treatment of 500 ng/ml Diphtheria toxin to eliminate remaining tumor cells - Further purification with FITC-BS1-B4-lectin by FACS	^166^
Lung carcinoma	- Mincing - Scalpel - 0.1 cm^3^	0.5 mg/ml Collagenase type I, 60 min, RT	Endothelial cell medium	Cell culture dish and Matrigel bed	- Isolate CD105+ cells by MACS	^167^
Renal carcinoma	- Mincing - Scissors	Collagenase type II (1 h, 37 °C)	MCDB131 medium, 10 ng/ml EGF, 1 µg/ml Hydrocortisone, bovine brain extract, 20% FCS	1% Gelatin or Endothelial cell attachment factor	- CD105+ cells by MACS - Identified with CD105, CD31, and VWF by immunofluorescence and FACS	^168^
Cancer-associated adipocyte (CAA)						
Breast cancer	Not used	250 U/ml Collagenase type I (30 min, 37 °C)	2D: DMEM, 10% FCS, 1% P/S 3D: DMEM, 25 mM glucose, 10% FBS, 1% P/S	2D: 6 well plate 3D: 6 or 4.2 mg/ml fibrin matrix	- 200 µm cell strainer - Wash with Krebs-Ringer Bicarbonate buffer for purification - Identified with Bodipy 493/503 by immunofluorescence	^169^
Breast cancer	- Mincing	1 mg/ml Collagenase I (1 h, 37 °C)	DMEM/F12, 10% FBS	Cell culture plate	- 100 µm cell sieve - Centrifugation	^170^
Omentum cancer	- Mincing - Scissors, scalpel - mm size	0.2% Collagenase I (1 h, 37 °C)	DMEM/F12 (1:1), 1% Penicillin, 1% Streptomycin, 0.1% BSA	Cell culture flask	- Filter using nylon mesh - Identified with Bodipy 493/503 by immunofluorescence	^171^
Tumor pericyte						
Hemangioma	- Mincing, homogenize - Scalpel, pestle - 2 mm^3^	50 µg/ml LiberaseTM + 5 U/ml Dispase (40–50 min, 37 °C)	EGM2-medium, 10% hiFBS, 1% GPS, EGM-2 Single Quot supplements except hydrocortisone	1 µg/cm^2^ FN-coated cell culture dish	- 100 µm cell strainer - Isolate GLUT1- CD31- PDGFRβ+ cells by MACS	^172^
Colon carcinoma	- Mincing	Collagenase IA + Collagenase II + Collagenase IV + DNAse I (15–20 min, 37 °C)	*No cultivation*	*No cultivation*	- 70 µm cell strainer - Isolate CD45- CD31- NG2 + CD140b+ cells by FACS	^173^
CRC/Lung cancer/Glioblastoma	- Cutting - Scissors	1.5 mg/ml Collagenase I + 1.5 mg/ml Collagenase II (40-60 min, 37 °C)	*No cultivation*	*No cultivation*	- Isolate NG2+ cells by MACS	^174^

*bFGF* basic fibroblast growth factor, *CD3* CD3 molecule, *CX3CR1* C-X3-C motif chemokine receptor 1, *DAPI* 4′ 6-diamidino-2-phenylindole, *DDR2* discoidin domain receptor tyrosine kinase 2, *DMEM* Dulbecco’s modified eagle medium, *ECad* E cadherin (CDH1), *EDTA* ethylenediaminetetraacetic acid, *EGF* epidermal growth factor, *EGM2-MV* endothelial cell growth medium MV2, *EpCAM* epithelial cell adhesion molecule, *FACS* fluorescence-activated cell sorting, *FAP* fibroblast activation protein alpha, *FBS* fetal bovine serum, *FCS* fetal calf serum, *GPS* guinea pig serum, *hEGF* human epidermal growth factor, *hFGF* human fibroblast growth factor, *hiFBS* heat inactivated fetal bovine serum, *HMB45* premelanosome protein (PMEL), *IL6* interleukin 6, *KRT* keratin, *Ly6G* lymphocyte antigen 6 family member G, *M-CSF* macrophage colony stimulating factor, *MHCII* major histocompatibility complex, class II (HLA-DR), *NG2* neural/glial antigen 2, *P/S* penicilin/streptomycin, *PDGFRα* platelet derived growth factor receptor alpha, *PECAM1* platelet and endothelial cell adhesion molecule 1 (CD31), *R3-IGF-1* long Arg3 insulin like growth factor 1, *RPMI1640* Roswell park memorial institute, *S100A4* S100 calcium binding protein A4, *VEGF* vascular endothelial growth factor, *VIM* vimentin, *VWF* von willebrand factor, *αSMA* alpha smooth muscle actin.

### Tissue acquisition

Tumor tissue samples from donors, including animal models and tumor patients, are collected through biopsy or surgical resection. Once the tissue specimen has been obtained from the donor, the sample is immediately immersed in a tissue preservation solution (transport medium), such as Hank’s balanced salt solution (HBSS), to avoid tissue necrosis or the initiation of apoptosis. The specimen is transported and stored at low temperature to preserve tissue integrity for further procedures.

### Tissue dissection

After tissue acquisition, the specimen is dissected to remove unwanted components, such as necrotic or nontumor regions, and minimize contaminants. Tissue dissection ensures the exclusion of extraneous tissues by isolating specific regions of interest and reducing contamination from nontarget cell types. This step is particularly important when working with tumor samples to ensure that the isolated primary cells are derived from tumor tissue^68^.

### Tissue disaggregation and dissociation

Dissociation is required to isolate individual cells. This procedure aims to isolate cells from the surrounding ECM or neighboring cells in preparation for subsequent cell expansion or analysis. Three main approaches are used for tissue dissociation: mechanical dissociation, enzymatic digestion, and chemical dissociation^69^. The three methods can be used independently or in combination during the dissociation process.

#### Mechanical dissociation

Mechanical dissociation is a simple approach for physically disrupting tumor tissues. This procedure entails tissue fragmentation with scissors, scalpels, and homogenizers routinely employed to disintegrate tissue samples. There are two major approaches: (1) tissue explant and culture of cells released from the fragmented tissue and (2) cell expansion using mechanical dissociation alone^70^. The tissue explant method involves simply culturing small tissue pieces in culture dishes and collecting cells that have migrated out of the pieces. This method decreases tissue size and reduces the risk of cell loss in later stages, such as the filtration of floating cells or enzymatic digestion^71^. The tissue explant method could provide additional benefits by preserving communication between outgrowing cells and tissue fragments and supplying cytokines or growth factors derived from tissue fragments^72^. However, cell outgrowth from explant tissues requires a relatively longer time to harvest cells, and usually, this method selectively isolates cell types with enhanced migratory functions^72^.

Another method using mechanical dissociation alone involves collecting the floating cells released from the loosened tissue fragments after the mechanical disruption of tissues^73,74^. Mechanical dissociation is generally followed by filtration or additional purification to remove remaining tissue fragments and separate the cell suspension for subsequent cell expansion or analysis. Although this approach can eliminate cells during filtering and purification steps, it is advantageous because this quick and simple method can be used for obtaining single viable cells^75,76^. Therefore, mechanical dissociation is advantageous because it is a straightforward method that does not introduce additional biological or chemical factors that could impact cell viability or phenotype. However, the outcomes of mechanical dissociation might be inconsistent and can be influenced by the skills of the individuals conducting the procedure^77^.

#### Enzymatic digestion

Enzymatic digestion is a widely employed method in which enzymes disintegrate tissues into individual cells. Cells embedded within a tissue adhere not only to ECM proteins but also to neighboring cells. Enzymatic approaches can be effective at disrupting cell adhesion and liberating cells from tissue^70^. The selection of enzymes for enzymatic digestion should be based on careful consideration of the isolated cell tissue type, target cell type, and downstream applications. Enzymatic digestion is usually combined with mechanical disaggregation to reduce the tissue size and increase the surface area accessible for enzyme action^78,79^. The use of this tandem strategy reduces the reaction time and increases cell yields^80^.

ECM-degrading enzymes have been frequently used to remove ECM macromolecules and encourage cell detachment. *Collagenase* is the most widely employed enzyme that breaks down native collagen, a prevalent ECM protein in connective tissues^81^. Dispase is also a suitable enzyme for degrading ECM proteins, especially collagen type IV and fibronectin. As a neutral protease, dispase is relatively less cytotoxic than other antimicrobial agents and prevents cell clumping without causing damage to cell membranes^82,83^. In addition, hyaluronidase is often used to degrade hyaluronic acid (HA), which is a ECM protein abundant in cancer tissues^84^. A variety of ECM-degrading enzymes, including elastase and Liberase, are available. While ECM-degrading enzymes primarily degrade ECM components, trypsin generally disrupts cell junctions and detaches cells completely from the tissue. Trypsin can effectively separate cell clumps into individual single cells, but it can also induce damage to proteins on cell membranes^85^. Deoxyribonuclease I (DNase I) often combines with other enzymes during enzymatic digestion. When cells dissociate from tissue, the free DNA released from dead cells can become entangled, impair proteolysis, and induce cell reaggregation. By combining DNase I with other enzymes, avoiding unwanted cell clumping caused by free DNA is possible^83^.

In addition to well-known enzymes, a commercial tumor dissociation kit from Miltenyi Biotec utilizes enzymatic digestion methods^86^. Although the dissociation kit guarantees that essential cell surface epitopes can be preserved, it does not explicitly disclose the specific enzyme components included. Thus, anticipating the potential biological effects of these enzymes on the primary cells of interest and their consequences is challenging.

Due to the efficacy of enzymatic digestion in yielding greater quantities of cells and diverse cell populations from tissues, it has been employed for decades as a method for isolating primary cells. Although enzymes with distinct specificities are advantageous for obtaining a single-cell suspension, they have detrimental effects on critical cell surface proteins and cell viability, depending on the circumstances. Therefore, identifying the optimal conditions for enzymatic digestion for the sample is crucial.

#### Chemical dissociation

In addition to proteolytic enzymes or glycosidases, chemical agents are also used to weaken cell-cell and cell-ECM interactions while isolating primary cells. Various cations, including calcium, potassium, and sodium, play essential roles in maintaining cellular integrity and cell adhesion. Chemical agents such as ethylene diamine tetraacetic acid (EDTA) and ethylene glycol tetraacetic acid (EGTA) are frequently utilized to perturb ion-dependent adhesion between cells and the ECM by chelating cations^39,87^. These agents are typically combined with trypsin to break intercellular bonds. Loosening these connections allows primary cells to be released and isolated from tissues^88^.

### Incubation and cell expansion

#### Culture platform

After the tissue specimen is disaggregated, fragmented pieces or single cells isolated from the tissue are incubated for primary cell outgrowth and expansion. In many previous studies, primary cells have been isolated and cultured for expansion using tissue culture plates that provide a hydrophilic, attachable surface for anchorage-dependent cells^89,90^. In some studies, plastic dishes coated with ECM proteins such as collagen, laminin, and basement membrane proteins were often used to promote cell adhesion, growth, and differentiation^91^. However, conventional monolayer cell culture conditions cannot provide an in vivo mimetic microenvironment because of the absence of the natural structure of tumors and the presence of ECM proteins that constitute the TME^92^.

#### Culture media

Proper cell culture media supplemented with essential nutrients and growth factors is required to support the in vitro proliferation and maintenance of primary cells. Serum is one of the key components that provides growth factors, hormones, lipids, and minerals for cell culture. The most common serum used in cell culture includes fetal bovine serum (FBS), also known as fetal calf serum (FCS), and bovine calf serum (BCS). While various cytokines and growth factors are supplied by neighboring cells in the TME, these factors should be supplemented in culture media for proper cell growth and maintenance. For example, basic fibroblast growth factor (bFGF) and epidermal growth factor (EGF) are used to culture fibroblasts and endothelial cells, respectively^93,94^.

Chemical compounds are introduced into the culture media. In the case of macrophage culture, the antioxidant 2-mercaptoethanol, also known as β-mercaptoethanol, is supplemented to reduce oxidative stress by removing free radicals^91,95^. In addition, antibiotics are frequently used during tissue acquisition, processing, and culture to prevent contamination as primary cells are directly isolated from human or animal tissues, and there are frequent sources of microbial contamination, such as commensal flora and subclinical infections^85,96^. The antibiotics frequently used in primary cell isolation and culture include penicillin, streptomycin, amphotericin B, gentamicin, and kanamycin. These supplementary components and the selection of appropriate basal media are crucial. Because the composition of each basal medium varies in terms of inorganic salts, amino acids, vitamins, glucose/carbohydrates, buffering agents, and other components, selecting suitable media formulations based on the specific cell type is important.

### Purification and separation of specific cell types

To isolate a specific cell type, adequate purification and separation steps are essential. Cell type purification can be performed before and after cell incubation and expansion. Cell type-specific characteristics such as cell shape, size, and surface proteins should be defined to separate target cell types from heterogeneous cell mixtures. The most common cell purification and separation techniques for cancer include magnetic-activated cell sorting (MACS) and fluorescence-activated cell sorting (FACS)^97,98^.

MACS is an immunomagnetic cell separation method that relies on the surface proteins of cells^99,100^. Magnetic beads are conjugated with antibodies, lectins, or enzymes. They are used to tag target cells that express surface protein markers. The magnetic bead–cell complexes are transferred to a column, and target cells labeled with magnetic beads can be captured and separated by applying a magnetic field to the column. Target cells are further recovered, and the pure population of interest can be harvested. FACS is another approach in which cell type-specific markers are used^101,102^. Unlike in MACS, target cells are labeled with fluorophore-conjugated antibodies. When the labeled cells are passed through a laser beam, they are sorted according to fluorescence intensity and scattered light. MACS and FACS separate cells based on cell type-specific protein markers and can be utilized for both the positive and negative selection of target cells.

Additional approaches involve using the physical features of cells. Density gradient centrifugation is a technique for separating cells based on the physical properties of cells. The most common method is Percoll gradient centrifugation^87,103^. Percoll is a density gradient medium that contains particles with specific physical characteristics. By using this medium, a density gradient can be produced. While the Percoll gradient remains stable, cells of differing densities settle into the specific bands corresponding to their density upon centrifugation. While Percoll centrifugation improves cell viability, this purification method can induce massive cell loss^87^.

Cell culture media is another method for purifying and obtaining specific cell types. Selection media are specialized media that support the selective growth of the desired cell population while minimizing the growth of unwanted cells or contaminants^104,105^. The composition of the selection media is optimized by adjusting the concentration of various components to achieve the desired selectivity, selective media for various cell types are also commercially available. As several strategies exist to separate specific cell populations, determining the optimal conditions for obtaining highly viable and pure target cells is essential. After collecting the cells of interest, characterizing and authenticating the isolated primary cells are necessary to determine their origin, purity, viability, and other characteristics.

## Challenges and potential breakthroughs in primary cell isolation

### Phenotypic alterations induced by the isolation and culture of primary cells

Primary cells have long been utilized for in vitro research, especially in oncology, because they maintain patient-specific characteristics and can be further utilized for clinical research and precision medicine^106^. As we discussed in the previous section, there are numerous combinations of methods available for isolating and expanding target cells in vitro. Consequently, the isolation process for the same cell type differs among individual studies, and there are no consensus protocols for cell type-specific isolation. However, some previous studies have reported that the phenotype of primary cells is affected by the isolation process and in vitro culture conditions. Nichols et al. examined two different approaches to compare the impact of the isolation method on primary tendon cells: tissue explant culture and enzymatic digestion^107^. The two isolation processes substantially affected cell morphology, proliferation, and marker expression. Tendon cells isolated by the explant method exhibited a more activated and myofibroblast-like phenotype, while those isolated by enzymatic digestion exhibited an altered phenotype not observed in vivo. These results indicate that different isolation methods critically affect cell behavior and phenotype.

**Fig. 4:**
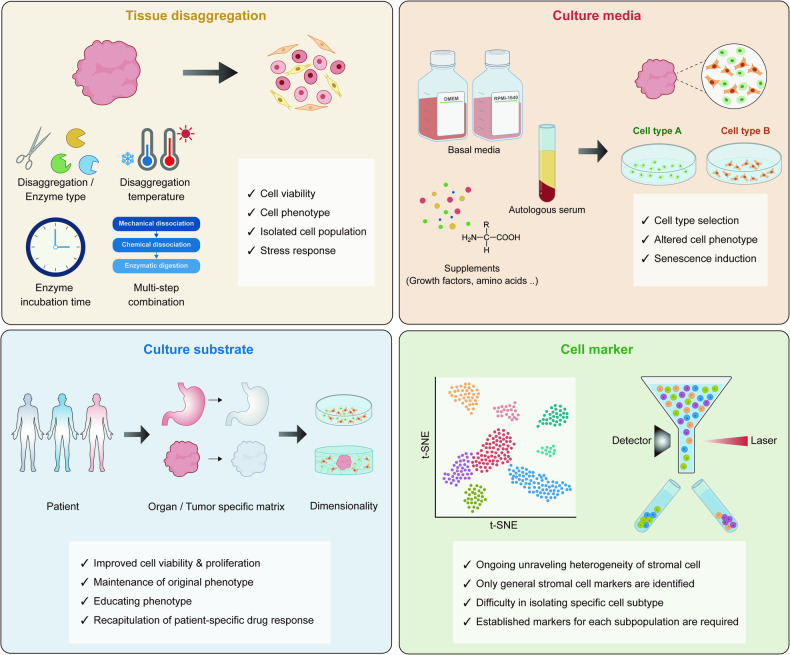
Possible impacts of each isolation step on primary cells. Summarized illustration showing how primary isolation procedures affect primary cells. Tissue disaggregation methods, cell culture media, cell culture substrates, and cell type selection markers should be carefully selected to minimize the deleterious effects of deconstruction.

In addition to the isolation process, in vitro culture itself changes the cell phenotype. For example, human primary fibroblasts isolated by the general method, which includes enzymatic digestion, cell expansion on a substrate-coated plastic plate, and further purification by MACS, were utilized to assess the effect of established culture conditions^108^. This research indicated that merely culturing primary fibroblasts under common conditions could induce the differentiation of fibroblasts into myofibroblast-like phenotypes, regardless of the media composition or whether serum was included. Given that the sequential steps, from primary cell isolation to cultivation, impact the population and characteristics of isolated cells, carefully designing all the procedures to achieve the desired isolation of specific cell types while preserving their inherent characteristics is crucial. In the following section, we investigate the influence of each factor on the isolation and culture of primary cells, with a primary focus on enzymes, culture media, and culture platforms (Fig. 4, Table 3).

**Table 3:** Impact of each isolation step on primary cells.

Process	Target	Condition	Findings	Ref
Disaggregation	Fibroblasts (lung tissue)	Enzyme type and incubation time 1) Enzyme type: Liberase DL/TL/TM/Collagenase P 2) Incubation time: 15 min/60 min	• Shorter incubation time lower protease-strength enzyme (Liberase) → Insufficient to isolate stromal cells → High yield of CD45+ immune cells • Longer incubation time with Collagenase P → A great diversity of cell types isolated → Low yield of CD45+ immune cells → Higher yield of CD45- EpCAM- CD31- CD90+ cells (fibroblasts)	^111^
Disaggregation	Single-cell suspension (breast and ovarian cancer)	Enzyme type and incubation temperature 1) *B. licheniformis* serine protease, 6 °C 2) Collagenase + Hyaluronidase, 37 °C	• Stress response induced by dissociation with Collagenase/Hyaluronidase at 37°C • Stress response-associated genes include *FOS, FOSB, ATF3*, and *HSP* • Upregulation of MHC-class I related genes by Collagenase/Hyaluronidase-based enzymatic digestion	^112^
Disaggregation	Microglial cell and astrocyte (brain tissue, glioblastoma)	Disaggregation type 1) Mechanical disaggregation (MD): Dounce homogenizer, 4 °C 2) Enzymatic disaggregation (ED): Collagenase + DNase, 37 °C	• Alterations in gene and protein expression induced by ED • Upregulation of immediate early genes by ED • ED lowers the yield of microglia detected by classical markers (CD45^low^/CD11b + ) • Cells isolated by ED were smaller in size	^110^
Disaggregation	Tumor lymphocytes (cutaneous T-cell lymphoma)	Combination of MD and ED 1) Medimachine (automated mechanical disaggregation) 2) Collagenase 1 A + Medimachine	• Decreased cell viability in the sample processed with Medimachine alone • Higher cell yields with collagenase pretreatment • Infiltrating T lymphocytes isolated with the collagenase pretreatment	^109^
Disaggregation	Single-cell suspension (gut mucosa)	Multistep dissociation 1) One-step: Collagenase (37 °C) 2) Two-step: EDTA (37 °C) - Collagenase (37°C) 3) Three-step: EDTA (4 °C) - Protease (4 °C) - Collagenase(37 °C)	• Balanced recovery of multiple cell types was achieved by one-step dissociation • Multistep protocols support the isolation of specific types (e.g., Three step protocols for epithelial cells)	^175^
Culture media	Tumor epithelial cells (PDAC)	Cell line media and Organoid media 1) Minimal media 2) Cell line media (RP10) 3) Organoid media (OWRNA)	• Cell state shift induced by culture media • PDAC cell line and organoid in the reciprocal media condition → Cell line lost scBasal features while Organoid gained scBasal expression	^176^
Culture media	Mammary epithelial cells	Media type 1) MCDB 170: serum-free media 2) WIT-P: serum-free media 3) M87A: low stress media	• Media type is important for the maintenance of lineage heterogeneity and cell growth • Rapid senescence and loss of heterogeneity induced by MCDB 170 • Impressive expansion of luminal cells in passage 2 and 3 caused by WIT-P • Maintenance of multiple lineages and robust growth supported by M87A	^115^
Culture media	Endothelial cells	Media type 1) Vascular cell basal Media (ATCC) 2) VascuLife Basal Media (LifeLine Celltech) 3) EC Basal Media (Lonza) 4) EC Media MV (PromoCell) 5) Human microvasc. EC basal Media (Cell applications) 6) Endopan MV microvasc. EC Basal Media (PAN-Biotech) 7) MCDB 131 Media (Gibco)	• Find optimal commercial media for successful primary cell isolation • Using MCDB131 medium failed to isolate pure and propagating endothelial cells • Isolation success and behavior of primary endothelial cells depend on the culture medium and the composition or nature of supplements	^114^
Culture media	Macrophages	Essential supplements 1) RPMI 1640 2) DMEM 3) MEM 4) McCoys 5a 5) IMDM	• Consider essential components for primary cell maintenance and function in vitro • Macrophages cultured in DMEM that lack nonessential amino acid (NEAA) → Smaller in size, less densely packed → Upregulated expression of *TNFα, mLILRB1, and sLILRB1* → Impaired function of macrophage • NEAA supplementation in DMEM restores DMEM-induced changes in macrophages	^116^
Culture media	HNSCC and CRC cancer tissue slices	Use patient-derived autologous serum (AS)	• Using AS induces a balanced induction of signal transduction pathways • Enhanced preservation of phenotypic and molecular features of patient tumors • Increased probability of predicting patient-specific chemotherapy outcomes	^118^
Culture platform	HNSCC and CRC cancer tissue slices	Patient-specific matrix 1) Patient tumor-derived matrix protein (TMP) cocktail coating 2) Gelatin coating 3) Collagen coating 4) Matrigel coating	• Tumor explants cultured on patient-specific TMP coating show improved maintenance of tissue morphology, proliferation, and cell viability • Noncoating: lost tumor architecture, decreased viability and proliferation, decreased activation of oncogenic pathways • Gelatin coating: similar to noncoated well • Collagen coating: only supports tumor proliferation • Matrigel coating: only improved cell viability	^118^
Culture platform	Mesenchymal stromal cells	The dimensionality of culture substrate 1) 2D explant culture 2) 3D explant culture using PLMatrix	• The 3D explant method increased cell yields • 3D PLMatrix provides matrix stiffness close to soft tissue (~0.1 kPa) • Cells isolated by 3D explant have the potential to differentiate into three lineages (adipogenic, chondrogenic, and osteogenic lineage) and maintain cell type-specific markers	^122^
Culture platform	Skeletal muscle, skin, and liver cells	Tissue-specific ECM 1) Muscle ECM coating 2) Skin ECM coating 3) Liver ECM coating 4) Collagen coating 5) Noncoating	• Tissue-specific ECM supports improved cell proliferation and the maintenance of cell type-specific phenotype	^177^
Culture platform	Endothelial colony-forming cells (Colon tumor)	Tumor-specific ECM 1) Fibrin gel 2) Normal ECM gel 3) Tumor ECM gel	• Tumor-specific ECM supports in vitro cells to mimic tumor-associated phenotypes • Tumor ECM promotes the formation of tumor-like vasculature • Tumor ECM includes additional components that are not present in normal ECM (e.g Fibronectin, Periostin, Versican, Thrombospondin-2, and Tenascin)	^178^
Culture platform	Monocyte/Macrophage (Ovarian cancer)	Cell phenotype educated by ECM 1) Decellularized tissue slide of low disease tissue (LD) 2) Decellularized tissue slide of high disease tissue (HD) 3) Tissue culture plate	• Tumor ECM alters the macrophage transcriptome • In vitro, ECM educated-Tumor associated macrophages (TAMs) show gene expression profiles of TAMs found in human tissue	^179^

*ATF3* activating transcription factor 3, *ECM* extracellular matrix, *EpCAM* epithelial cell adhesion molecule, *FOS* Fos proto-oncogene, *AP-1* transcription factor subunit, *FOSB* FosB proto-oncogene, *AP-1* transcription factor subunit, *HSP* heat shock protein, *mLILRB1* leukocyte immunoglobulin-like receptor B1, *PDAC* pancreatic ductal adenocarcinoma, *scBasal* single-cell Basal, *sLILRB1* leukocyte immunoglobulin-like receptor B1, *TNFα* tumor necrosis factor-alpha.

### Enzyme effects

Enzymatic digestion is an inevitable step in detaching cells from tissue and making cell suspension. The types of enzymes used, along with conditions such as temperature and duration of treatment, significantly impact primary cells. The following changes in primary cells induced by enzymatic disaggregation have been identified: changes in cell yield, cell viability, isolated cell population, phenotype, and gene expression patterns.

As previously noted, enzymatic degradation of ECM proteins and cellular adhesions is critical for optimizing cell yield. For tumor lymphocyte isolation, the efficacy of mechanical disaggregation via Medimachine, an automated mechanical disaggregation instrument, was evaluated in conjunction with collagenase application^109^. Collagenase pretreatment followed by mechanical disaggregation improved cell viability and cell recovery. In addition, atypical lymphocyte populations that were highly infiltrative were also recovered by the combination method. Although enzyme treatment improved cell viability in these experiment, some conflicting studies have shown that enzymatic digestion induces cell death in specific cell populations, such as neurons and astrocytes, in brain tissue^110^. These inconsistent results imply that enzymatic digestion may enhance the isolation of viable cells with high yields of specific cell types. However, enzymes can also inflict cellular damage and have variable separation efficiency across different cells.

Depending on the cell type, isolating particular cells from tissues may be difficult; therefore, the isolation process must vary based on the target cells. For example, Waise et al. examined disaggregation enzymes and incubation times to explore the optimal conditions for isolating fibroblasts from primary human tissues^111^. Treatment with enzymes with lower proteolytic activity, such as Liberase, for shorter incubation time was insufficient for isolating stromal cells; however, this treatment also yielded a greater proportion of CD45+ immune cells. A longer incubation time with the collagenase P enzyme cocktail improved the diversity of the cell types while decreasing the proportion of CD45+ immune cells. In addition, the latter approach yielded a significantly greater proportion of fibroblasts. This study demonstrated that the types of enzymes employed and the duration of enzyme treatment should vary depending on the desired target cell type.

The optimal temperature for determining enzyme activity varies depending on the type of enzyme. The enzyme type and treatment temperature are coupled during enzymatic digestion. For instance, while collagenase and hyaluronidase are active at significantly higher temperatures, a serine protease from *B. licheniformis* exhibited activity at 6 °C. A notable difference in transcription was observed between the two enzyme-based dissociation methods^112^. The expression of stress response-associated genes, such as Fos proto-oncogene (*FOS*), FosB proto-oncogene (*FOSB*), activating transcription Factor 3 (*ATF3*), and heat shock protein (*HSP*), as well as major histocompatibility complex (MHC) class I-associated genes, was upregulated by the combination of collagenase and hyaluronidase at 37 °C. Like in previous findings, enzymatic digestion at higher temperatures led to the upregulated expression of specific genes, especially immediate early genes (IEGs)^110^. As *FOS* and *FOSB* are also categorized as IEGs, we can deduce that enzymatic digestion at elevated temperatures induces a shared transcriptional bias for certain genes. These altered gene expression patterns may indicate phenotypic changes in primary cells that could influence subsequent downstream experiments and analyses. Therefore, being aware that enzyme treatment can alter the expression of particular genes is crucial. Subsequently, during further analysis or experiments, assessing whether any biases were introduced by the enzymes is imperative.

### Media effects

Owing to the intricate composition of cell culture media, determining the individual influence of each component on primary cells is a formidable challenge. Despite this complexity, pursuing optimal media formulations is crucial for the effective isolation and expansion of primary cells to promote cellular proliferation while maintaining intrinsic phenotypic attributes. Numerous studies have shown the impact of media formulations on primary cell growth, viability, morphology, gene expression patterns, and overall functionality^113–117^.

There are many typical culture media, such as Rosewell Park Memorial Institute (RPMI)-1640 medium and Dulbecco’s modified essential medium (DMEM), and they vary in terms of the composition and concentration of amino acids, vitamins, inorganic salts, and glucose. Leopold et al. studied seven types of commercially available primary endothelial cell isolation media^114^. Complex media containing endothelial cell growth supplements (ECGS) facilitated cell proliferation and viability, while defined media, which included specific growth factors such as EGF, FGF2, and VEGF, enhanced cell outgrowth and in vitro angiogenesis. In addition to the defined medium MCDB-131, other types of media enabled the isolation of endothelial cells without affecting the phenotype. Recognizing that using suboptimal culture media may result in the failure of primary cell isolation is important. Therefore, identifying an appropriate media formulation specific to the type of target cell is paramount.

Another crucial aspect is identifying the essential elements required for the expansion of target cells. For example, the cultivation of macrophages in DMEM, which lacks nonessential amino acids (NEAAs), altered the phenotype of macrophages^116^. The cell size was much smaller, and the expression of specific genes encoding tumor necrosis factor alpha (*TNFα*) and leukocyte immunoglobulin-like receptor subfamily B member 1 (*mLILRB1* and *sLILRB1*) was upregulated without NEAAs. In addition to the alterations in morphological and transcriptional phenotypes, functional changes in macrophages were also observed. The release of soluble proteins, which is one of the major functions of immune cells, was impaired by using DMEM, and this effect was restored when NEAA was supplemented with DMEM.

Accurate in vitro emulation of patient-specific tumor-stroma interactions is contingent upon preserving the phenotypic and functional attributes of primary cells. If such characteristics are altered during the isolation and expansion phases, the resulting tumor model does not represent the biological complexities inherent in the patient’s original tumor. In this context, several methodologies have been proposed to enhance the preservation of tumor-specific features during in vitro cultivation. For example, supplementation of patient-derived autologous sera (AS) in culture media could be an alternative to the generally used animal-derived serum and supplementary factors^118^. Since AS reflects the enrichment level of growth factors that varies between individuals, it induced a balanced induction of signaling pathways, and the phenotypic and molecular features of the patient’s tumor were better preserved. The AS supplement also improved the prediction of patient-specific outcomes for chemotherapy. Patient-derived samples such as serum samples are generally difficult to obtain and limited in quantity; however, they are appealing and indispensable materials for reconstructing real tumors de novo.

### Culture platform

Many researchers have attempted to improve in vitro cell culture techniques, particularly those mimicking the intricate in vivo microenvironment. The ECM is a known pivotal determinant of cellular behavior and phenotype^119–121^. Nevertheless, most studies focusing on the isolation and utilization of primary cells have predominantly employed two-dimensional (2D) monolayer cultures.

The environmental cues used in monolayer culture using plastic dishes differ substantially from those cues in in vivo tumor niches. Unlike in in vivo conditions where stromal cells are embedded within the ECM and actively engage with its proteins, monolayer cultures offer only a planar surface devoid of supportive ECM components. This discordance may lead to alterations in many aspects of primary cells. Two main approaches have been recently demonstrated to improve primary cell isolation and expansion: tissue-specific ECM and 3D explant-based cell isolation.

Utilizing tumor-specific ECM for primary cell expansion would facilitate the maintenance of the tumor-associated phenotypes of stromal cells. For example, Majumder et al. utilized four coating materials: gelatin, collagen, Matrigel, and patient tumor-derived matrix protein (TMP) cocktail^118^. While other coating materials supported only tumor proliferation or cell viability, TMP supported tumor proliferation, viability, and the maintenance of tumor morphology. By engineering a personalized ecosystem with TMP, the conservation of tumor characteristics was enabled during ex vivo tissue culture. Although TMP was used for tissue cultivation in this study, it could also be a promising material for isolating and expanding patient-specific stromal cells while preserving their original phenotype.

In addition to the specific ECM proteins, the dimensionality of the culture platform is also important for mimicking an in vivo-like ecosystem. Recent advances have included the implementation of 3D explant-based approaches for primary cell isolation. For instance, Egger et al. utilized a 3D human platelet lysate matrix to isolate primary mesenchymal stem/stromal cells (MSCs) by embedding adipose tissue^122^. MSCs isolated in this manner exhibit specific surface markers and can differentiate into adipogenic, chondrogenic, and osteogenic lineages. The 3D explant technique offers a more physiologically relevant environment for primary cell isolation. In contrast to those in conventional 2D methodologies, cells that migrate from tissue explants embedded in a 3D explant model integrate into a matrix that not only is enriched in ECM proteins but also mimics the mechanical properties of soft tissue, resulting in more effective retention of their original phenotypes.

### Purification markers

Numerous studies have isolated primary cells from mixed cell populations by leveraging cell type-specific markers. Cell separation techniques such as FACS and MACS rely on the expression of marker proteins to segregate target cells. However, the recent recognition of stromal cell heterogeneity — facilitated by advancements in single-cell technologies — reveals that previous studies may have inadvertently selected markers without accounting for cellular subtypes, thereby leading to biased isolation of subpopulations. For example, Huang et al. purified CAFs from tumor tissues using FAP alone as a CAF marker^123^. However, the pancancer single-cell analysis revealed that CAFs can be classified into six main subpopulations: myoCAFs, inflaCAFs, adiCAFs, EndMT-CAFs, PN-CAFs, and apCAFs^48^. Among those subsets, FAP can only identify inflaCAFs. Thus, the findings from previous studies utilizing FAP+ CAFs might only be applicable to inflaCAFs.

In addition, different studies have often defined identical cell types using divergent molecular markers. This inconsistency complicates our understanding of cell behaviors and functions, as it conflates the characteristics of stromal cells identified through different markers as traits of a single, homogenized cell type. Consequently, further research is imperative for elucidating stromal cell heterogeneity. Future investigations should prioritize the utilization of a broad array of subtype-specific markers for the isolation and functional characterization of stromal cells.

## Perspectives

We have reviewed stromal cell heterogeneity and strategies for isolating primary cells from patient-derived tissues, including their potential effects on primary cells. Although the tumor stroma plays a key role in the TME by dictating the behavior of tumor cells, most previous studies focused only on developing more advanced tumor cell-based models and neglected the importance of the tumor stroma. Current tumor models only partially incorporate human stromal cells or employ heterologous stromal counterparts for tumor modeling. Furthermore, even though stromal cells are known to be composed of heterogeneous populations, many studies use stromal cells without defining which subtypes they are. Although integrating all stromal cell types and subtypes into in vitro tumor models is challenging, certain cells are critical because they are found in all tissues and play a key role in shaping the microenvironment. These cells include fibroblasts and macrophages^124^. For instance, ASPN+ fibroblasts and SPP1+ macrophages are notably prevalent near tumors in various cancer types^125^. Their intense interactions with adjacent tumor cells contribute to immune exclusion within tumors by creating a desmoplastic niche, a factor that is closely associated with poor prognosis. These examples highlight the fact that certain subtypes are strongly linked to patient prognosis and treatment response.

Patient-derived explants (PDEs) are a viable alternative that mirror, to a certain extent, the stromal constitution of the original tumor microenvironment. This ex vivo culture of patient tumor specimens helps preserve the inherent stromal components and microarchitectural features, thereby maintaining critical tumor-stroma interactions. This highlights the utility in evaluating therapeutic responses and determining pharmacodynamic biomarkers. However, the temporal preservation of tissue integrity within PDEs is ephemeral, and the absence of their expandability limits their use in low-throughput applications and diminishes reproducibility^126^. Thus, humanized tumor models that accurately display the heterogeneity of tumor cells and stromal cells are urgently needed to better represent patient tumors for the development of precision medicine.

Advancements in tumor modeling now allow for detailed replication of both cancer cells and their complex surroundings, thanks to improved methods for breaking down and reassembling tumors. These methods include advanced sequencing and metabolomics for analyzing tumor cells and sophisticated techniques for studying the noncellular environment, such as ECM.

In vitro tumor reconstruction harnesses engineering methodologies to emulate the in vivo TME. Scaffolds, particularly those derived from decellularized ECM, offer a physiologically congruent 3D architecture for cell culture that supports cell adherence and facilitates cell-ECM interactions^127^. These scaffolds are particularly conducive to organoid culture and promote in vivo-like cellular dynamics. Additionally, to recapitulate complex cell-cell interactions within tumors, coculture systems incorporating stromal and cancer cells are utilized, underscoring the necessity of maintaining stromal cell integrity throughout in vitro processes^128,129^. The spatial arrangement of cellular constituents is addressed through innovative technologies such as 3D bioprinting and tumor-on-a-chip technology, with the goal of accurately reconstructing the intricate in vivo tumor architecture^130,131^.

After reconstruction, the fidelity of in vitro tumor models compared to their in vivo counterparts was ascertained through comprehensive multiomics analyses and immunohistochemical imaging, through which the in vitro constructs were compared with the original tumor samples. Such rigorous validation and ongoing refinement of deconstruction and reconstruction techniques are pivotal in generating robust, humanized in vitro tumor models, and will promote in vitro studies to clinical translation for precision medicine and drug discovery.

To develop a reliable humanized tumor model, we could consider a reconstructed tumor model as a potential platform that can accurately replicate heterogeneous human stroma. However, achieving such an ideal in vitro reconstruction poses challenges because the prerequisites need to be addressed. First, the specific functions and molecular markers of stromal cell subpopulations should be clearly defined across various cancer types. Many studies have obtained information about the diverse subpopulations of stromal cells through single-cell analysis. However, within a given cancer type, there is no consensus regarding the types and characteristics of these subpopulations. This information is imperative during tumor destruction, as it enables the isolation of desired stromal cell subtypes, comprehension of stromal cell attributes, and ultimately, the utilization of these cells.

Second, optimal protocols for primary cell isolation should be established according to stromal cell subpopulations. The isolation process, from disaggregation to cell expansion, may result in biased acquisition of specific cell populations or phenotypic alterations of isolated cells. Understanding stromal cell subtypes and standardizing isolation protocols for each cell subset should be performed to acquire cell populations of interest.

Third, the spatial distribution of stromal cells should be considered when evaluating tumor reconstruction. Tissue deconstruction inevitably accompanies the loss of information about cellular locations within the TME. As the spatial location of stromal cells determines the cell-cell and cell-ECM signals to which cells are exposed, the position of stromal cells in their original locations is crucial. In recent years, single-cell spatial technologies such as spatial transcriptomics have emerged; these methods help researchers understand the correlations between phenotypes and the locations of cells and characterize cell-cell interactions that affect tumor behavior and therapeutic responses. Locational information provided by spatial technologies can be further used to ensure the optimal distribution of stromal cells in the reconstructed tumor model.

Upon successfully developing an authentic humanized tumor model, this platform could serve as a potent preclinical model for investigating therapies targeting the stroma. The limitations of traditional preclinical tumor models, which often lack a complex human stromal component, have impeded the translation of promising therapeutic strategies into effective human cancer treatments. For instance, a preclinical study demonstrated the efficacy of saridegib, a Smoothened (SMO) inhibitor pivotal for the hedgehog pathway, in diminishing stromal compartments within tumors and increasing the density of blood vessels within the tumor core by targeting CAFs. These outcomes collectively contributed to a reduction in overall tumor size and an extension of survival in mice^132,133^. However, despite these promising results, saridegib failed to demonstrate therapeutic advantages in *phase I* and *phase II* clinical trials in humans^134,135^. This failure might be attributed to disparities between murine and human stromal responses. Reproducing a fully humanized ecosystem through the use of a reconstructed tumor model will minimize the disparities between in vitro and in vivo settings and effectively mimic biological events that occur in vivo. This deconstruction and reconstruction approach may provide a more precise representation of therapeutic responses, thereby facilitating the identification of the most appropriate therapeutic approach for each individual.
